# Discovery of 5-Methylthiazole-Thiazolidinone Conjugates as Potential Anti-Inflammatory Agents: Molecular Target Identification and In Silico Studies

**DOI:** 10.3390/molecules27238137

**Published:** 2022-11-22

**Authors:** Michelyne Haroun, Anthi Petrou, Christophe Tratrat, Aggeliki Kolokotroni, Maria Fesatidou, Panagiotis Zagaliotis, Antonis Gavalas, Katharigatta N. Venugopala, Nagaraja Sreeharsha, Anroop B. Nair, Heba Sadek Elsewedy, Athina Geronikaki

**Affiliations:** 1 Department of Pharmaceutical Sciences, College of Clinical Pharmacy, King Faisal University, Al-Ahsa 31982, Saudi Arabia; 2 School of Pharmacy, Aristotle University of Thessaloniki, 54124 Thessaloniki, Greece; 3 Division of Infectious Diseases, Weill Cornell Medicine, New York, NY 10065, USA; 4 Department of Biotechnology and Food Science, Faculty of Applied Sciences, Durban University of Technology, Durban 4000, South Africa; 5 Department of Pharmaceutics, Vidya Siri College of Pharmacy, Off Sarjapura Road, Bangalore 560035, India; 6 Department of Pharmaceutical Sciences, College of Pharmacy, AlMaarefa University, Dariyah, Riyadh 13713, Saudi Arabia

**Keywords:** thiazole, thiazolidinone, anti-inflammatory activity, PASS, COX-1, COX-2, LOX, docking

## Abstract

A series of previously synthesized 5-benzyliden-2-(5-methylthiazole-2-ylimino)thiazoli- din-4-one were evaluated for their anti-inflammatory activity on the basis of PASS predictive outcomes. The predictive compounds were found to demonstrate moderate to good anti-inflammatory activity, and some of them displayed better activity than indomethacin used as the reference drug. Structure–activity relationships revealed that the activity of compounds depends not only on the nature of the substituent but also on its position in the benzene ring. The most active compounds were selected to investigate their possible mechanism of action. COX and LOX activity were determined and found that the title compounds were active only to COX-1 enzymes with an inhibitory effect superior to the reference drug naproxen. As for LOX inhibitory activity, the derivatives failed to show remarkable LOX inhibition. Therefore, COX-1 has been identified as the main molecular target for the anti-inflammatory activity of our compounds. The docking study against COX-1 active site revealed that the residue Arg 120 was found to be responsible for activity. In summary, the 5-thiazol-based thiazolidinone derivatives have been identified as a novel class of selective COX-1 inhibitors.

## 1. Introduction

**Figure 1:**
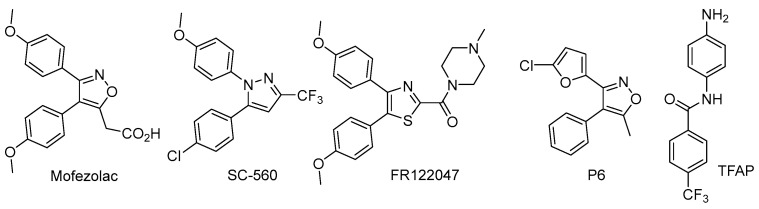
Representative COX-1 selective inhibitors.

Non-steroidal anti-inflammatory drugs (NSAIDs) are a heterogeneous group of chemical compounds that differ in their antipyretic, analgesic and anti-inflammatory effects. They act mainly through the inhibition of cyclooxygenase (COX), a key enzyme in the first prostanoid biosynthetic pathway and divided into the non-selective, cyclooxygenase (COX)-1 and -2 inhibitors and the selective COX-2 inhibitors. COX inhibitors are among the most widely prescribed drugs for the treatment of inflammatory conditions. On the other hand, classic NSAIDS are associated with a variety of side effects such as nausea, vomiting, constipation, headache, diarrhea, allergic reactions and rash. Hypertension, heart attacks and heart failure can also be associated with their use. The most important side effects of COX-1 are gastric ulcers and bleeding, but also renal failure [1,2], while drugs of the new generation of NSAIDs, selective COX-2 inhibitors were associated with increased risk of myocardial infarction and cardiovascular thrombotic events. For example, the drugs Rofecoxib and Valdecoxib associated with the occurrence of severe cardiovascular events have been withdrawn from the market [3,4]. However, it has been demonstrated that the gastric concern of NSAIDS was not related to solely COX-1 inhibition but requires both COX-1 and COX-2 inhibition [5]. In fact, gastric lesions were observed when a combination of both selective COX-1 inhibitor and selective COX-2 inhibitor was used. Moreover, evidence supports that COX-1 inhibition alone failed to induce gastric ulceration [6]. In addition, COX-1 selective inhibition up-regulated COX-2 expression in gastric mucosa resulting in increasing prostaglandin E2 production to a level necessary for mucosal integrity [6]. To date, only a few selective COX-1 inhibitors have been identified as promising anti-analgesic and anti-platelets agents without causing gastric lesions including Mofezolac, SC-560, FR122047, P6 and TFAP (Figure 1) [7]. Among them, Mofezolac is the only drug marketed in Japan for clinical use as potent pain killer. 

Unlike COX-2, COX-1 as molecular target for other diseases remains less explored. Few reports showed the implication of COX-1 enzyme in many other diseases such as cancers [8], neuro-inflammation [9,10], thrombosis [11]. For instance, several ovarian cancer cells demonstrated overexpression of COX-1 enzyme without any detection of COX-2 expression [12] and a COX-1 selective inhibitor showed high potency against COX-1-expressing human ovarian adenocarcinoma (OVCAR-3) cells [13]. SC-560 presented promising inhibitory action against colon cancer cell and showed cell cycle arrest in Go/G1 phase [14]. Mofezolac was also reported to display in vivo anti-tumoral activity for colon cancer [15]. Furthermore, COX-1 may play a key role in protecting the heart in acute myocardial infarction with reperfusion [16]. On the basis of the information above, COX-1 appears to be an attractive molecular target for developing new effective molecules as anti-inflammatory, anticancer, antiplatelet agents that can reduce gastric toxicity. 

**Figure 2:**
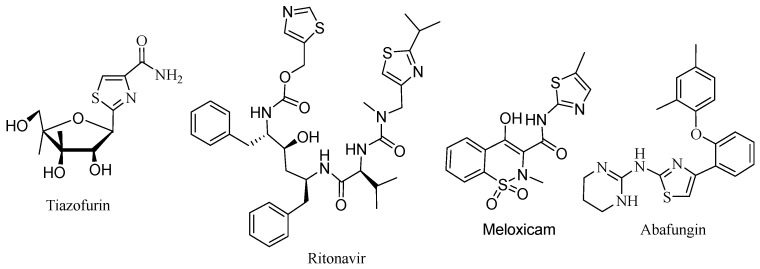
Approved drugs with thiazole scaffold.

Thiazole ring was found to possess a significant broad spectrum of pharmacological and pharmaceutical actions in the biological system. Among them are anti-inflammatory [17,18,19,20], antimicrobial [21,22], anticancer [23,24,25], anti-HIV [26,27], antioxidant [28,29], antidiabetic [30,31], local anesthetic [32] activities, among many others [28,33,34]. Furthermore, thiazole scaffold is present in some FDA-approved drugs, such as tiazofurin [35], an antineoplastic drug, vitamin B1 [36], ritonavir, an approved drug against HIV [37], meloxicam [38] NSAID and abafungin, a broad-spectrum antifungal agent with a novel mechanism of action for the treatment of dermatomycoses (Figure 2). 

**Figure 3:**
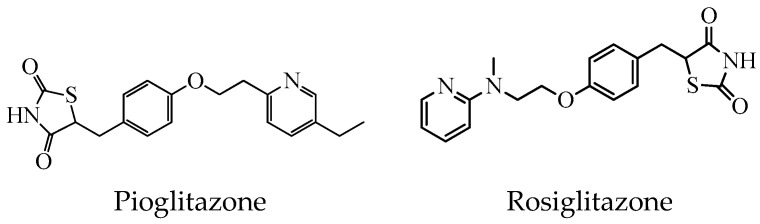
Approved drugs with thiazolidinone scaffold.

On the other hand, thiazolidinone core possesses many different pharmacological activities such as antimicrobial [39,40,41,42], anti-inflammatory [43,44,45,46], anti-diabetic [47,48,49], anticancer [41,42,50], anti-HIV [51,52], antioxidant [53] and carbonic anhydrase inhibitory potency [54,55]. Pioglitazone and rosiglitazone (Figure 3), used to treat type II diabetes, are also very important thiazolidinone-containing agents approved for human use. 

One of the key approaches in drug design is molecular hybridization based on the combination of two or more pharmacophores of different biologically active molecules in the frame of one single molecule [56,57]. The aim of this approach is mainly to improve the activity profile and reduced undesired side effects. 

Thus, several thiazolidinone derivatives were designed and synthesized by introducing different arylidene substituents at the 5 position of the thiazolidinone moiety which, according to our previous observations [58], can be useful to encompass certain physico-chemical properties such as hydrophobic and steric. The aim of this research is to investigate the anti-inflammatory activity in vivo on carrageenan-induced mouse paw edema as well as in vitro cyclooxygenase and lipoxygenase inhibition of previously synthesized compounds [59,60] by the incorporation of two pharmacophores thiazole and thiazolidinone moieties in the frame of one molecule. To this aim, PASS prediction and molecular modelling simulation were both undertaken in order to select potential candidates for biological evaluation.

## 2. Results

### 2.1. Prediction of Spectra of Biological Activity

Biological activity prediction for fifty-four chemical structures was carried out using PASS [61]. This program was successfully used in the search for COX/LOX inhibitors earlier [62]. Each predicted biological activity for a particular compound is characterized by the Pa value, which reflects the probability of belonging to the class of “actives”. This program allows to classify compounds as “actives” and “inactives”. The higher the Pa value is, the more chance there is to confirm the activity in the experiment. 

Based on the prediction results (Table 1) we selected sixteen compounds with the highest Pa values for experimental testing of biological activity. They include compounds **16**, **5**, **8**, **7**, **6**, **14**, **13**, **4**, **17**, **15**, **11**, **3**, **2**, **10**, **9** and **12**. Pa values for the selected compounds varied from 0.360 (compound **12**) to 0.581 (compound **16**).

We should note that the probability *Pa* primarily reflects the similarity of the structure of a given molecule to the structures of molecules of the most typical active compounds in the corresponding subset of the training set. Thus, as a rule, there is no direct correlation of the values of *Pa* with quantitative activity characteristics [63]. Compound **1** was chosen as a “control” since *Pa* values are known to reflect the probability of presence of activity, but not the magnitude of activity.

As one may see from Table 1, several series with diverse substituents at position 5 of thiazole ring, such as hydrogen, methyl, phenyl, adamantyl, isothiazole, benzothiazole and benzoisothiazole, have been investigated for their potential anti-inflammatory activity with PASS program. The substituent in position 5 of thiazole ring strongly impacts the anti-inflammatory activity where the presence of methyl group in this position provided good prediction with *Pa* values ranging from 0.233 to 0.581.

### 2.2. Docking Studies

Based on encouraging outcomes from PASS results and in order to strengthen our prediction, we screened these fifty-four compounds in silico for their anti-inflammatory activity against the molecular targets COX-1, COX-2 and LOX enzymes using the same parameters (Table 1). 

For the docking studies, the most widely used enzymes in the literature, COX-1 and COX-2 in complex with ibuprofen and inhibitor SC-558, respectively, were selected (PDB code: 1EQG and 1CX2) and LOX (PDB code: 6N2W). At first stage, a validation test was performed to certify the program’s reliability. Firstly, the co-crystallized ligands were extracted and docked back into the corresponding binding pockets to determine the ability of AutoDock to reproduce the orientation and position of the inhibitor observed in the crystal structure. According to docking results, the orientations of the docked inhibitors were very close to that found in the crystal structure (Figure 3). 

It was observed from our predictive docking simulation that 5-metylthiazole series demonstrated better docking scores against COX-1 drug target. As for COX-2 and LOX, they demonstrated weak molecular interactions. In addition, some of these chosen compounds (**8, 13** and **16**) had better molecular interaction against COX-1 than both reference drugs naproxen and ibuprofen indicating, their potentiality to be considered as anti-inflammatory agents. Therefore, on the basis of PASS and molecular docking prediction, the 5-metylthiazole-based thiazolidinone derivatives were selected to investigate their anti-inflammatory activity. 

### 2.3. Chemistry

**Scheme 1:**
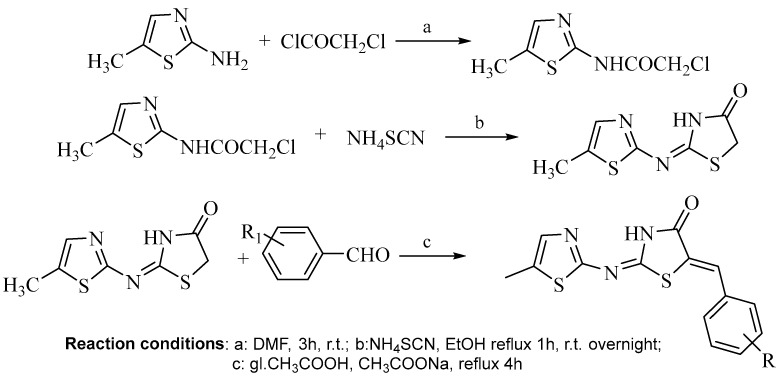
Synthesis of titled compounds.

Compounds were synthesized according to the general method described in our previous papers [59,60] and presented in Scheme 1.

In brief, the final products were successfully synthesized from commercially available 2-amino-5-methylthiazole which was treated with chloroacetyl chloride to obtain its corresponding amide derivative. The latter underwent cyclization in the presence of ammonium thiocyanate in refluxing ethanol affording 2-(5-methylthiazol-2-yl) thiazolidin-4-one and then heating to reflux with appropriate aldehydes to provide 5-benzyliden-2-(5-methylthiazol-2-ylimino)thiazolidin-4-ones. Overall, the reactions proceeded smoothly with good yields. 

### 2.4. Anti-Inflammatory Activity Assessment

#### Effect on Carrageenan-Induced Mouse Paw Oedema

For the study of possible anti-inflammatory action, the swelling caused in the sole of the right hind limb by intradermal administration of carrageenan was used as a model of inflammation [63]. Carrageenan-induced edema is a non-specific inflammation that results from a number of different transmitters and resembles human acute inflammation [63]. This type of edema is very sensitive to non-steroidal anti-inflammatory drugs and therefore carrageenan is considered a useful tool for the initial study and investigation of the possible effects of new anti-inflammatory agents.

The results of anti-inflammatory activity evaluation of the compounds together with reference drug, indomethacin, are given in Table 2 as percent inhibition of weight gain in the sole of the right hind leg muscle, compared to the left used as a reference.

As shown in Table 2, all the investigated compounds induced protection against carrageenan-induced mouse paw edema, showing moderate to good anti-inflammatory activity. The protection compounds ranged up to 57.8 %, while the reference drug, indomethacin, exhibited 47% protection at the same molar concentration. Four compounds **13**, **16**, **12** and **8** presented a promising anti-inflammatory profile with of 57.8% ± 1.3%, 57.6% ± 1.4%, 55.6% ± 1.5% and 55.4% ± 1.5% inhibition of edema, respectively. Compounds **1, 4, 6** and **10** displayed higher or equipotent activity than indomethacin. The remaining compounds showed lower activity. Compound **3** with hydroxyl group in para position showed the lowest potency (31.4%).

The study of structure–activity relationships revealed that the activity of the tested compounds depends on the substituent and its position at the benzene ring. Thus, the presence of 2,3-di-Cl, 3-Br, 4-Cl and 4-NO_2_ substitution at benzene ring was the most beneficial to the activity. Taking into account the biological statistics data, it can be observed that derivatives **8**, **12**, **13** and **16** are all equipotent, indicating that fluorine, chlorine and nitro substituents were found to be optimal for potency.

Shifting one of the chlorine atoms from position 3 (**13**) to position 4 or 6 (**14, 15**) significantly decreased the activity, while the introduction of 3-Br substituent (**16**) instead of 4-Br (**17**) notably increased the activity. Replacement of 4-Br by 4-Cl substituent (**12**) lead to improvement of activity. As for the nitro series, the derivatives **7** (3-NO_2_) and **8** (4-NO_2_) were found to be equipotent, while the derivative **6** (2-NO_2_) showed the same order of magnitude in activity as the reference drug. The presence of the electron donating group was found to be less favorable to activity, such as compounds **2**, **3**, **4** and **5**. However, the introduction of the hydrophobic methyl group to the derivative **3** (4-OH) leading to methoxy compound **4** was found to markedly increase the activity. In view of these results, it can be concluded that the hydrophobic nature of the substituent strongly positively influences the anti-inflammatory activity, while the electronic effect of substituent was insignificant to the activity.

### 2.5. Molecular Target Identification

The most common molecular targets for the action of anti-inflammatory agents are COX-1, COX-2 and LOX. Cyclooxygenase catalyzes the conversion of arachidonic acid to prostaglandins, important inflammatory mediators, also significant for gastric mucosa protection, platelet aggregation and kidney function. COX-1 is constitutively expressed, while COX-2 expression is inducible by inflammatory stimuli leading to increased prostaglandin release. Lipoxygenases (LOs) are a family of iron-containing enzymes that catalyze the deoxygenation of polyunsaturated fatty acids in lipids. Lipoxygenases have attracted the interest of the scientific community, as they are involved in the biosynthesis of leukotrienes that play an important role in the pathophysiology of several inflammatory and allergic diseases [64].

On the basis of the promising anti-inflammatory activity of our compounds, we decided to prospect the probable mechanism of action. From Table 1, COX-1, COX-2 and LOX were selected to predict whether the potential candidates demonstrate some ability to form favorable interactions with the investigated targets. This study revealed that our compounds were predicted to have better molecular interaction with only COX-1 target. In order to verify our prediction, the best three compounds (**8**, **13** and **16**) were selected to determine the inhibitory activity against the studied drug targets.

The effect of three active compounds as well as that of ibuprofen and naproxen, used as reference compounds, (concentration 200, 50, 25, 10, 0.1 μM) on both COX isoforms have been examined while nordihydroguaiaretic acid (NDGA) was used as reference for the inhibitory action against soybean LOX (Table 3). 

According to the in vitro assays (Table 3), ibuprofen and naproxen are better inhibitors of COX-1 than COX-2 as reported in the literature [63]. The examined compounds also appeared to be potent inhibitors of COX-1, in general better than naproxen. The most potent compound as COX-1 inhibitor was found to be compound **16** with 3-Br substitution on benzene ring, followed by compounds **13** and **8.** Moreover, no inhibition activity of our compounds was observed against COX-2 enzyme. As for LOX activity, the three tested compounds were found to exhibit weak inhibitory activity in comparison with the reference compound NDGA. In view of these findings, this study was in accordance with our predictive molecular docking approach, where COX-1 has been identified as the main molecular target for the anti-inflammatory activity of our compounds.

### 2.6. Docking Studies

In order to explain the inhibition profile of the title compounds, molecular docking studies were performed against COX-1 enzyme and the results are given in Table 4.

**Figure 4:**
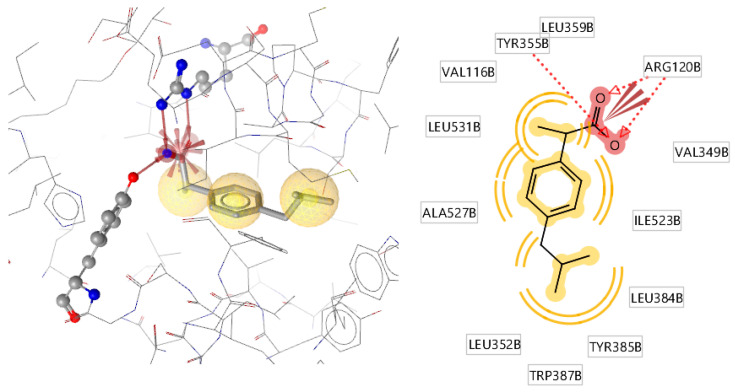
Docking pose of co-crystallized ligand, ibuprofen (S) (IBF), in the COX-1 active site. Red dotted arrows indicate H-bond and yellow spheres indicate hydrophobic interactions.

**Figure 5:**
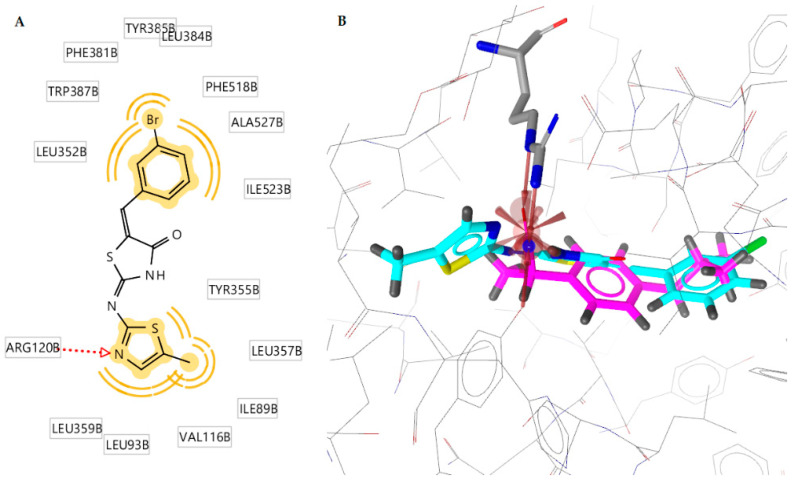
(**A**) 2D diagram of compound **16** in the binding pocket of COX-1. (**B**) Docked compound **16** (light blue) superimposed on the reference drug naproxen (magenta) in the active site of COX-1 enzyme. Red dotted arrows indicate H-bond and yellow spheres indicate hydrophobic interactions.

According to in silico studies the co-crystallized ligand, ibuprofen (S), binds to the COX-1 active site forming three hydrogen bonds through its carboxylate group, two with Arg120 and another one with Tyr355 (Figure 4). Moreover, hydrophobic interactions with the residues Val116, Val349, Ala527, Ile523, Leu384, Tyr385, Tyr384, Leu352 and Leu531 were detected. The most active compound **16** binds to COX-1 enzyme in a similar way to ibuprofen by establishing a hydrogen bond with residue Arg120, throughout its nitrogen atom of thiazole moiety. Moreover, compound **16** was predicted to have hydrophobic contacts with plenty amino acid residues such as Tyr355, Leu375, Leu93, Ile89, Tyr358, Leu357 and others, which stabilize further the complex enzyme compound and justify its low IC_50_ value (1.08 μM) (Figure 5, Table 4).

## 3. Materials and Methods

κ-Carrageenan and lipoxygenase type I-B from soybean were purchased from Sigma (St. Louis, MO, USA). COX inhibition was estimated using the “COX Inhibitor Screening Assay” kit (Cayman Chemical Co., Ann Arbor, MI, USA). For the in vivo experiments, female and male (23–30 g) mice R”’ were kept in the Centre of the School of Veterinary Medicine (EL54 BIO42), Aristotelian University of Thessaloniki, which is registered by the official state veterinary authorities (presidential degree 56/2013, in harmonization with the European Directive 2010/63/EEC). The Animal Ethics Committee of the Prefecture of Central Macedonia (no. 270079/2500) approved the experimental protocols.

### 3.1. Prediction of Biological Activity Spectra by PASS

PASS Online is available on the Internet allowing to predict over 4000 types of biological activity based on a structural formula [65,66]. It has many advantages [67]. PASS method and examples of its application are described in detail elsewhere [68].

### 3.2. Effect on Carrageenan-Induced Mouse Paw Oedema

The animals were weighed and divided into control, standard and test groups. Each group contained 10 mice. The first group of mice was treated with 0.1 mL of saline intraperitoneal (control), second group was administered with a dose of 10 mg/kg of the suspension of indomethacin (standard) and the tests groups were treated with an equimolar dose of the suspension of test compounds relative to standard drug. An aqueous solution of carrageenan 0.1 mL, 1% *w*/*v*) was injected subcutaneously to the sub-plantar region of the right hind paw, with the left paw as control. The tested compounds (suspended in water with a few drops of Tween 80) were given *i.p*. (0.15 mmol/kg) 5 min before the carrageenan administration. After 3.5 h, the hind paws were excised and were weighed separately. The produced oedema was estimated as a paw weight increase [69].

### 3.3. Inhibition of COX-1 and COX-2 Activity

The effect of compounds on COX-1 and COX-2 inhibition was measured using a commercial kit provided by Cayman (Cayman Chemical Co., Ann Arbor, MI), applying the instructions of the manufacturer. The kit uses ovine COX-1 and human recombinant COX-2 enzymes. This assay is an excellent tool which can be used for general inhibitor screening, or to eliminate false positive leads generated by less specific methods. The assay measures PGF_2a_ produced by SnCl_2_ reduction of COX-derived PGH_2_. The prostanoid product was quantified via enzyme immunoassay using a broadly specific antibody that binds to all the major prostaglandin compounds [69].

### 3.4. Inhibition of LOX Activity

The reaction mixture contained (final concentration 300 μM) the test compounds, dissolved in absolute ethanol (10–300 μM), or the solvent (control), soybean LOX, dissolved in 0.9% NaCl solution (250 u/mL) and sodium linoleate (100 μM), in Tris-HCl buffer, pH 9.0. The reaction was followed for 7 min at 28 °C, recording the absorbance (234 nm) of a conjugated diene structure, due to the formation of 13-hydroperoxy-linoleic acid. The performance of the assay was verified using NDGA as a reference. For the estimation of the type of inhibition, the above experiments were repeated, using 1 mM sodium linoleate, which is higher than the saturating substrate concentration [69].

### 3.5. Docking

Molecular modeling studies were performed using the software AutoDock 4.2 [70]. X-ray crystal structures of COX-1 (PDB code: 1EQG) [71], COX-2 (PDB code: 1CX2) [72], LOX (PDB code: 6N2W) with their corresponding bound inhibitors, were retrieved from Protein Data Bank (PDB). All procedures were carried out following our previously reported work [73]. 

In particularly, for the preparation of ligand structures, 2D structure was sketched in ChemDraw 12.0 and hydrogens were added and converted to mol2 format. The grid size was set to 50 × 50 × 50 xyz points with grid spacing of 0.375 Å. For the docking simulation, default values of quaternation, translation and torsion steps were applied. The Lamarckian Genetic Algorithm with default parameters was applied for minimization. The number of docking runs was 100. The graphical depictions of all ligand-protein complexes were achieved by Discovery Studio visualizer version 4.0 (BIOVIA, San Diego, CA, USA). 

To begin with, the co-crystallized ligands were docked into the active site of COX-1, COX-2 and LOX enzymes, respectively, to validate the accuracy of the docking program AutoDock 4.2. The results revealed that the docked ligands were exactly superimposed on the co-crystallized bound ones with a root mean square deviation value (RMSD) of 0.93 Å (COX-1), 0.35 Å (COX-2) and 0.68 Å (LOX) indicating the ratability of our docking protocol.

## 4. Conclusions

Fifty-four thiazole-based thiazolidinones, previously synthesized, were predicted for their biological activity spectra and docking studies. Seventeen compounds were chosen for biological evaluation according to the results of prediction (PASS and docking). The Pa values for the most promising compounds ranged from 0.455 to 0.581. For three equipotent compounds, Pa values ranged from 0.371 to 0.465, which also correspond to the top predictions. Thus, despite the absence of correlation between the Pa value and magnitude of the activity, PASS predictions allow us to optimize the selection of the compounds with favorable activity. Prediction reveals that these compounds could be considered as potential anti-inflammatory agents. Based on these results, candidate compounds were selected and evaluated for their anti-inflammatory activity. It was found that compounds provided protection up to 57.8% and the most promising compounds were 4-NO_2_ (**8**), 2,3-di–Cl (**13**), 3-Br (**16**) and 4-Cl (**12**), while four compounds (**1, 4, 6** and **10**) were equipotent with the reference drug. The hydrophobic character of the substituent on benzylidene ring was found significant for activity. The in vitro evaluation of COX-1/COX-2 and LOX inhibitory activity of three of the most active compounds revealed that they are potent COX-1 inhibitors with IC_50_ in the range of 1.08–14.38 μM, better than that of the reference compound Naproxen (IC_50_ = 40.10 μM), which was in agreement with results from our predictive molecular approach. Docking studies against COX-1 active site provided molecular insight into activity where the residue Arg 120 was found determinant for activity. In conclusion, 5-methylthiazole thiazolidinone conjugates were discovered as potent selective COX-1 inhibitors and could be regarded as novel anti-inflammatory agents. 

## Figures and Tables

**Table 1:**
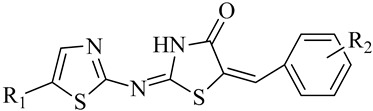
Anti-inflammatory PASS prediction and Docking assisted predicted values against COX-1, COX-2 and LOX.

No	R_1_	R_2_	Binding Free Energy (kcal/mol)			Pa	No	R_1_	R_2_	Binding Free Energy (kcal/mol)			Pa
			COX-1 (PDB: 1EQG)	COX-2 (PDB: 1CX2)	LOX (PDB: 6N2W)					COX-1 (PDB: 1EQG)	COX-2 (PDB: 1CX2)	LOX (PDB:) 6N2W	
**1**	5-CH_3_	H	−7.15	−4.11		0.233	**28**	H	4-NO_2_	−2.87	−3.67	−3.44	0.167
**2**	5-CH_3_	2-OH	−5.89	−2.40	−4.28	0.373	**29**	H	3-NO_2_	−3.66	−2.59	−2.54	0.250
**3**	5-CH_3_	4-OH	−6.10	−2.84	−6.19	0.375	**30**	H	4-N (CH_3_)_2_	−5.37	−3.64	−3.62	0.208
**4**	5-CH_3_	4-OCH_3_	−7.23	−6.58	−5.17	0.448	**31**	4-CH_3_	4-Cl	−7.54	−6.28	−4.25	0.301
**5**	5-CH_3_	2, 5-OCH_3_	−7.58	−7.56	−2.47	0.579	**32**	5-CH_3_	4-NO_2_	−5.31	−6.70	−2.43	0.276
**6**	5-CH_3_	2-NO_2_	−5.12	−4.28	−6.57	0.465	**33**	5-CH_3_	2-Cl	−7.46	−6.54	−1.28	0.178
**7**	5-CH_3_	3-NO_2_	−5.27	−4.32	−6.45	0.479	**34**	Ph	H	−3.61	−2.55	−1.85	0.223
**8**	5-CH_3_	4-NO_2_	−10.16	−5.27	−6.88	0.482	**35**	Ph	3-Cl	−4.37	−6.19	−4.69	0.175
**9**	5-CH_3_	3-F	−7.46	−5.94	−4.51	0.365	**36**	Ph	2-Cl	−4.12	−6.03	−5.02	0.156
**10**	5-CH_3_	4-F	−7.55	−6.34	−6.35	0.371	**37**	Ph	4-Cl	−5.80	−6.45	−4.25	0.114
**11**	5-CH_3_	2-Cl	−5.30	−5.66	−7.04	0.395	**38**	4-ad	4-Br	−5.66	−4.83	−5.13	0.155
**12**	5-CH_3_	4-Cl	−6.75	−6.52	−5.16	0.360	**39**	4-ad	4-N (CH_3_)_2_	−6.29	−5.17	−5.74	0.128
**13**	5-CH_3_	2, 3-Cl	−11.82	−6.50	−4.28	0.455	**40**	isothiazole	4-NO_2_	−5.20	−6.37	−4.82	0.202
**14**	5-CH_3_	2, 4-Cl	−7.89	−5.28	−1.60	0.463	**41**	isothiazole	4-OCH_3_	−6.01	6.00	−4.51	0.151
**15**	5-CH_3_	2, 6-Cl	−8.03	−6.24	−3.87	0.422	**42**	isothiazole	H	−5.91	−4.32	−6.71	0.077
**16**	5-CH_3_	3-Br	−9.54	−12.41	−5.64	0.581	**43**	isothiazole	Vanillin	−6.24	−6.86	−2.69	0.073
**17**	5-CH_3_	4-Br	−7.64	−8.45	−2.20	0.443	**44**	benzothiazole	4-NO_2_	−6.47	−5.19	−5.11	0.122
**18**	H	H	-	−5.28	−1.59	0.217	**45**	benzothiazole	2-Cl	−5.16	−5.40	−2.73	0.285
**19**	H	4-OH	−2.14	−4.16	−3.49	0.289	**46**	benzothiazole	3-Cl	−5.52	−5.63	−5.92	0.190
**20**	H	2-OCH_3_	−5.17	−4.50	−5.52	0.265	**47**	benzothiazole	4-Cl	−7.53	−5.60	−5.11	0.107
**21**	H	4-OCH_3_	−6.19	−5.20	−5.70	0.347	**48**	benzothiazole	4-OCH_3_	−6.10	−4.37	−2.73	0.204
**22**	H	3-OCH_3_, 4-OH	−6.48	−4.27	−6.57	0.366	**49**	benzothiazole	4-OH	−5.19	−5.53	−5.92	0.138
**23**	H	2, 5-OCH_3_	−5.33	−6.22	−6.03	0.362	**50**	benzoisothiazole	2-Cl	−6.16	−6.38	−4.60	0.147
**24**	H	2-Cl	−4.10	−3.57	−2.47	0.279	**51**	benzoisothiazole	3-Cl	−6.54	−6.20	−2.88	0.261
**25**	H	3-Cl	−5.32	−4.76	−2.01	0.285	**52**	benzoisothiazole	4-Cl	−6.53	−6.18	−2.36	0.163
**26**	H	4-Cl	−4.25	−4.83	−3.69	0.199	**53**	benzoisothiazole	4-NO_2_	−4.37	−3.15	−2.51	0.094
**27**	H	4-Br	-	-	−1.39	0.292	**54**	benzoisothiazole	4-OH	−7.93	−6.42	−5.63	0.122
**Naproxen**			−9.53	−8.11									

**Table 2:**
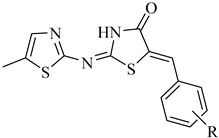
Anti-inflammatory activity of titled compounds.

A/A	R′	Oedema Inhibition ^α^, (%)	A/A	R′	Oedema Inhibition ^α^, (%)
**1**	H	51.6 ± 1.5 **	**10**	4-F	47.8 ±1.6 *
**2**	2-OH	43.1 ± 1.9 *	**11**	2-Cl	43.0 ± 1.7 *
**3**	4-OH	31.4 ± 2.3 **	**12**	4-Cl	55.6 ± 1.5 **
**4**	4-OCH_3_	48.3 ± 1.8 *	**13**	2, 3-Cl	57.8 ± 1.3 ***
**5**	2, 5-OCH_3_	44.9 ± 1.7 *	**14**	2, 4-Cl	35.2 ± 2.1 *
**6**	2-NO_2_	48.7 ± 1.8 *	**15**	2, 6-Cl	39.4 ± 2.3 **
**7**	3-NO_2_	54.5 ± 1.4 *	**16**	3-Br	57.6 ± 1.4 **
**8**	4-NO_2_	55.4 ± 1.5 **	**17**	4-Br	41.4 ± 1.9 *
**9**	3-F	34.8 ± 2.1 *	**Indomethacin**		47.0 ± 1.6 **

*** *p* < 0.0001; ** *p* < 0.0005; * *p* < 0.005, Student’s *t*-test.

**Table 3:** Activity effect of compounds on cyclooxygenase (COX) -1 and -2, and LOX.

No	Inhibition COX-1		Inhibition, COX-2	LOX
	Inhibition (%) 200 μM	IC_50_ (μM)	Inhibition (%) 200 μM	Inhibition (%) 100 μM
**8**	100	14.38 ± 0.7 **	0	7.88 ± 2.1 **
**13**	100	1.10 ± 0.3 *	0	37.2 ± 1.8 ***
**16**	100	1.08 ± 0.3 *	0	46.7 ± 1.5 **
**Ibuprofen**	68.0 ± 1.2 **	12.7 ± 0.5 *	46.0 ± 1.4 *	
**Naproxen**		40.10 ± 1.6 **	17.0 ± 2.3 **	
**NDGA**				94.0 ± 1.1 *

*** *p* < 0.0001; ** *p* < 0.0005; * *p* < 0.005, Student’s *t*-test.

**Table 4:** Docking results and interacting residues against COX-1.

Compound	Ovine COX-1 (PDB:1EQG)	
	Binding Free Energy (kcal/mol)	Hydrogen Bonds
**8**	−10.16	Arg120, Tyr355
**13**	−11.82	Arg120, Tyr355
**16**	−11.73	Arg120
**Naproxen**	−9.53	Arg120, Tyr355
**Ibuprofen**	−9.22	Arg120, Tyr355

## Data Availability

The data presented in this study are available on request from the corresponding authors.

## Sample Availability

Samples of the compounds are available from the authors.
